# Where is acetabular reaming concentrated during total hip arthroplasty for hip osteoarthritis? A computed tomography-based simulation analysis

**DOI:** 10.1007/s00264-026-06901-4

**Published:** 2026-06-24

**Authors:** Hiroto Funahashi, Yusuke Osawa, Hiroaki Ido, Takamune Asamoto, Yasuhiko Takegami, Shiro Imagama

**Affiliations:** 1 Department of Orthopedic Surgery, Nagoya University, Nagoya, Japan; 2 Department of Integrated Health, Nagoya University, Nagoya, Japan

**Keywords:** Total hip arthroplasty, Simulation analysis, Reaming, Developmental dysplasia of the hip, Primary osteoarthritis

## Abstract

**Purpose:**

The purpose of this study was to quantify the three-dimensional distribution of estimated acetabular bone requiring reaming during virtual cup placement in total hip arthroplasty (THA) for hip osteoarthritis (OA), providing a quantitative reference for direction-specific reaming patterns.

**Methods:**

In this CT-based simulation analysis, preoperative CT data from 118 female hips that underwent primary THA for primary OA or mild developmental dysplasia of the hip (DDH) between 2021 and 2024 were used to simulate cup placement at the true acetabulum. The pelvis was oriented to the functional pelvic plane, and the cup was positioned at 20° anteversion and 40° inclination. The simulated reamed area was quantified at 12 clock-face positions. Simulated reamed areas were compared by diagnosis using Holm-adjusted Student’s t tests and among positions using repeated-measures ANOVA with Holm adjustment.

**Results:**

The mean simulated reamed area was smallest at 1 o’clock (64 ± 45 mm^2^) and largest at 8 o’clock (137 ± 73 mm^2^) (*p* < 0.001). Post hoc comparisons showed larger reamed areas at 8 and 9 o’clock and smaller areas from 0 to 2 o’clock than at most other positions (*p* < 0.05). This posteriorly concentrated pattern was particularly evident in mild DDH, with smaller reamed areas from 0 to 2 o’clock and larger areas at 8 and 9 o’clock than at other positions.

**Conclusion:**

In this CT-based simulation study, simulated acetabular reaming was minimal in the anterosuperior region and greater posteriorly. These findings may provide anatomical information for understanding direction-specific reaming patterns, particularly in mild DDH.

**Supplementary Information:**

The online version contains supplementary material available at https://doi.org/10.1007/s00264-026-06901-4.

## Introduction

In cementless total hip arthroplasty (THA) for hip osteoarthritis (OA), achieving sufficient initial stability through appropriate press-fit promotes bony ingrowth on the cup surface [1, 2] and leads to favourable long-term outcomes [2, 3]. Acetabular reaming directly affects cup micromotion [4, 5]. Specifically, insufficient reaming may result in a relatively oversized cup, thereby increasing rim stress and causing perioperative periprosthetic fracture [6, 7], whereas excessive reaming may create a bone–implant gap, increasing micromotion and the risk of loosening [8, 9]. Acetabular reaming to achieve appropriate press-fit fixation of a planned cup size at the intended position is a technically demanding step in THA and has a substantial learning curve, particularly for surgeons early in their experience with the procedure [10, 11]. Therefore, three-dimensional understanding of which part of the acetabulum should be reamed and to what extent is important for appropriate acetabular reaming.

A thorough understanding of osteophyte formation and acetabular morphology is essential for appropriate acetabular reaming. Bombelli classified hip OA types based on osteophyte patterns on anteroposterior radiographs [12], and subsequent studies have focused on acetabular osteophytes, particularly rim osteophytes, in relation to impingement after THA [13, 14]. However, no study has quantitatively evaluated intra-acetabular osteophyte overgrowth and the corresponding amount of reamed bone. The extent and pattern of osteophyte formation may vary depending on the underlying disease leading to OA. Unlike in Western countries, where primary OA is the predominant indication for THA, OA secondary to developmental dysplasia of the hip (DDH) is more common in Japan and other Asian countries [15]. In DDH, OA develops in the setting of a shallow acetabulum and insufficient acetabular coverage [16], and acetabular morphology therefore differs fundamentally from that in primary OA [17]. Given these differences, the direction and extent of acetabular reaming anticipated during cup placement may also differ between DDH and primary OA. Preoperatively, surgeons use radiographs and computed tomography (CT) to estimate cup placement and bone removal. However, the three-dimensional distribution and extent of reaming within the acetabulum according to OA type and underlying diagnosis remain unclear. We hypothesized that quantitative assessment of three-dimensional reaming patterns of intra-acetabular osteophytes and subchondral bone may provide a quantitative anatomical reference for surgical education and improve the understanding of direction-specific acetabular reaming patterns. Therefore, using a simulation model in which the cup was virtually placed at the true acetabulum in patients with hip OA, we aimed to address the following study questions. (1) How is simulated acetabular reaming distributed across clock-face directions in the overall cohort? (2) Do simulated reamed areas differ by osteoarthritis type and underlying diagnosis? (3) Within each osteoarthritis type and underlying diagnosis, do simulated reamed areas differ among clock-face directions?

## Methods

### Patients

The ethics review board of our institution approved this study (approval no. 2020–0272). This study complies with the Declaration of Helsinki. This was a retrospective CT-based simulation study using preoperative CT data from patients who ultimately underwent primary THA. The simulation data were not used to guide the actual surgical procedure or intraoperative decision-making. A total of 463 hips in 424 patients underwent primary THA from January 2021 to December 2024.　Preoperative anteroposterior pelvic radiographs were obtained for all patients. Hips with a lateral center-edge (CE) angle < 25° and no other identifiable cause of OA were classified as DDH, while those with a lateral CE angle ≥ 25° and no identifiable cause of OA were classified as primary OA. Of these, 314 hips were excluded for the following reasons: prior hip surgery (92 hips), hip disorders other than primary OA or DDH (162 hips), male sex (19 hips), and the second-operated hips of patients who underwent bilateral THA (41 hips). Male patients were excluded because sex-related differences in acetabular morphology [18] and body size may influence cup size and the amount of reaming. In addition, 31 hips with a simulated cup CE angle < 0° were excluded, leaving 118 hips for the final analysis (Supplementary Fig. 1). Osteophyte morphology was classified on preoperative anteroposterior pelvic radiographs according to Bombelli’s classification [12] by two orthopaedic surgeons specializing in hip surgery. In cases of disagreement, the final classification was determined by consensus. Patient demographics, underlying diagnoses, and OA type classifications are summarized in Table 1. Race and ethnicity data were not collected because this information is not routinely recorded in our institutional database; therefore, we were unable to report participant race/ethnicity.

**Table 1:** Demographic data

		Diagnosis		OA type (Bombelli classification)			*p* value	
	Total (*N* = 118)	Primary OA (*N* = 30)	Mild DDH (*N* = 88)	Hypertrophic (*N* = 29)	Normal (*N* = 48)	Atrophic (*N* = 41)	Diagnosis	OA type
Age, years	67 ± 9	69 ± 6	66 ± 10	68 ± 7	67 ± 9	65 ± 10	0.109	0.284
Body mass index, kg/m^2^	25 ± 4	27 ± 3	24 ± 4	25 ± 4	26 ± 4	24 ± 5	0.005	0.332
Cup size, mm	49 ± 2	49 ± 2	49 ± 2	50 ± 2	49 ± 2	49 ± 2	0.357	0.117

*OA* Osteoarthritis, *DDH* Developmental dysplasia of the hip

### CT-based simulation

**Fig. 1:**
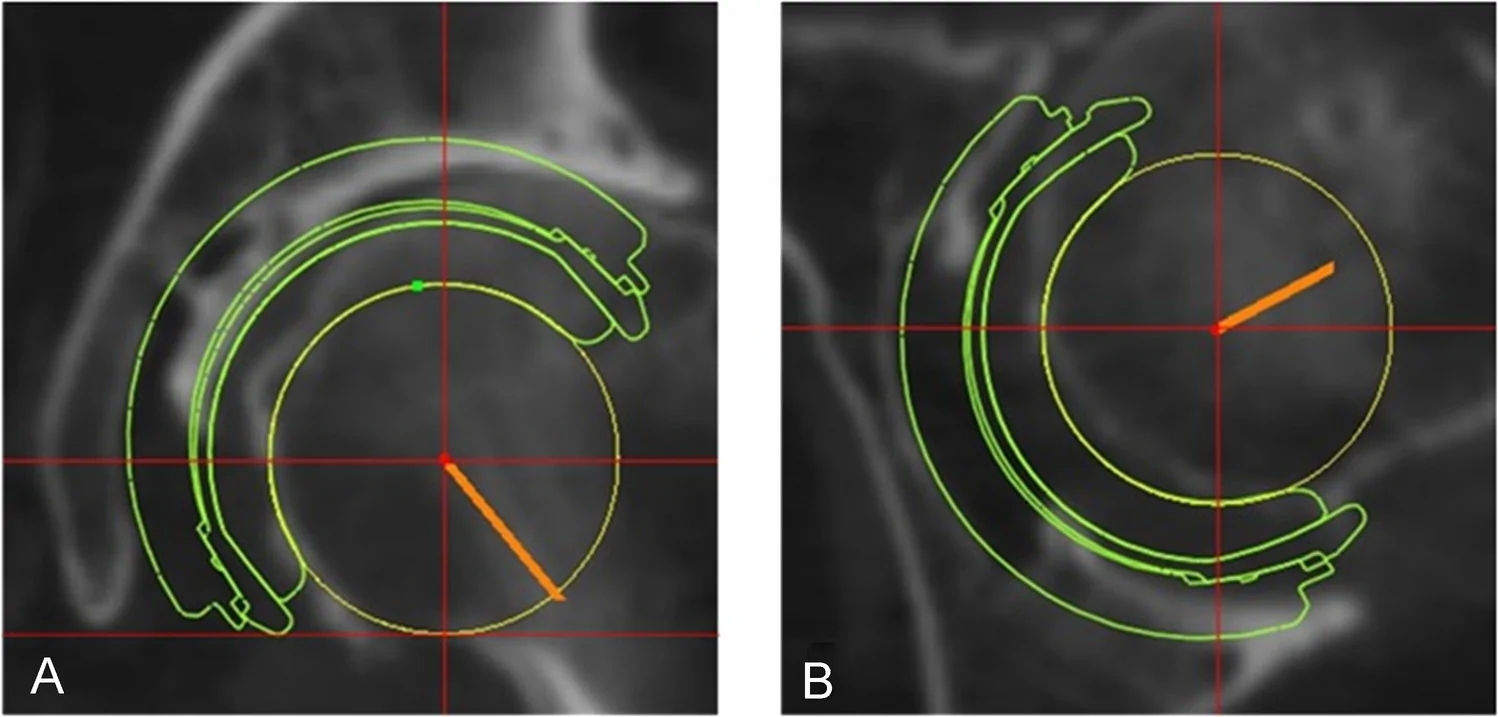
Virtual cup placement at the true acetabulum. Virtual cup implantation was performed in ZedHip on a pelvis oriented to the functional pelvic plane. The acetabular component was positioned at 20° of anteversion and 40° of inclination, with its inferior edge aligned with the transverse acetabular ligament and fitted to the rim of the true acetabulum (**A**). Cup size was selected as the best-fitting size on the axial slice passing through the cup centre (**B**)

**Fig. 2:**
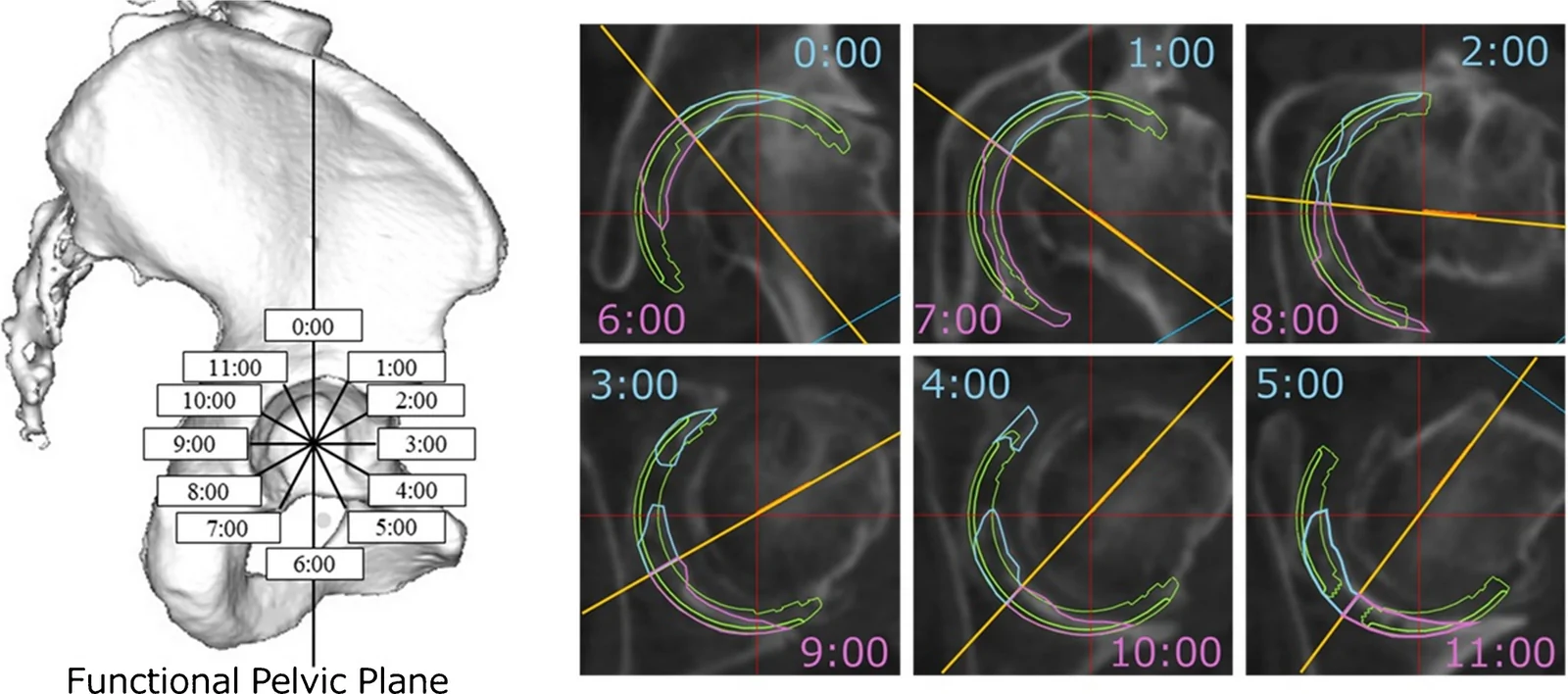
Clock-face analysis of the simulated reamed area within the acetabulum. In a pelvic coordinate system aligned with the functional pelvic plane, a clock-face diagram was created with the cup centre as the origin and the cranial direction defined as 0 o’clock, dividing the acetabulum radially into 12 sectors from 0 to 11 o’clock. The cup was advanced linearly along the insertion axis corresponding to the planned anteversion and inclination (orange line), and acetabular bone overlapping the outer surface of the cup along this path was defined as the reamed region on axial computed tomography images. Each panel shows the reamed region at a given clock position from 0 to 11 o’clock, and the simulated reamed area was quantified separately for each sector

All patients underwent preoperative CT scans from the superior iliac crest to the knee using a Toshiba Aquilion CT scanner (Toshiba Medical, Tochigi, Japan) with scanning parameters of 120 kVp, 320 mA, and a slice thickness of 1.0 mm. CT data were transferred to CT-based simulation software (ZedHip; Lexi Co. Ltd., Tokyo, Japan) for three-dimensional coordinate definition and implant placement simulation. The functional pelvic plane (FPP) was used as the pelvic coordinate system and was defined as the plane passing through both anterior superior iliac spines and parallel to the CT table in the supine position. The Trident® II acetabular cup (Stryker Orthopaedics, Mahwah, NJ, USA) was used for all simulations. Cup placement followed the method described by Yang et al. [19]. The virtual cup was seated within the peripheral rim of the true acetabulum to achieve rim fit, with the inferior edge aligned with the distal cotyloid notch, at the level of the transverse acetabular ligament. The cup was positioned at 20° of anteversion and 40° of inclination in the FPP coordinate system [20, 21]. The cup size was selected as the size that best matched the anteroposterior diameter of the native acetabulum on the axial CT slice passing through the planned cup centre (Fig. 1). After virtual cup placement, hips with a simulated cup CE angle < 0° were excluded because of the potential risk of inadequate cup fixation [22]. DDH hips in the final analysis that met the criterion of a cup CE angle ≥ 0° were defined as mild DDH. The reamed area was defined as the cross-sectional area of acetabular subchondral bone and osteophytes removed along the planned cup insertion axis and was quantified on the corresponding measurement plane for each clock-face sector. Measurement planes were defined using a clock-face coordinate system, with the cranial direction in the FPP pelvic coordinate system designated as 0 o’clock and numbers increasing toward the anterior pelvis (Fig. 2). Quantification was performed using ImageJ software (National Institutes of Health, Bethesda, MD, USA).

### Statistical analysis

Reliability was assessed using intraclass correlation coefficients (ICCs), and was excellent for both intraobserver and interobserver measurements, with ICCs ranging from 0.88 to 0.99 and 0.84 to 0.98, respectively. The mean of four measurements was used for the final analysis. Continuous variables were normally distributed by the Shapiro–Wilk test. Differences in reamed areas across clock-face directions were assessed using repeated-measures ANOVA with Greenhouse–Geisser correction when sphericity was violated, followed by Holm-adjusted paired t tests. Comparisons among OA types and between diagnoses were performed using one-way ANOVA or Student’s t tests, with Holm adjustment for multiple comparisons. Fisher’s exact test was used for categorical variables. Statistical significance was set at p < 0.05. Analyses were performed using EZR version 1.41 [23].

## Results

**Fig. 3:**
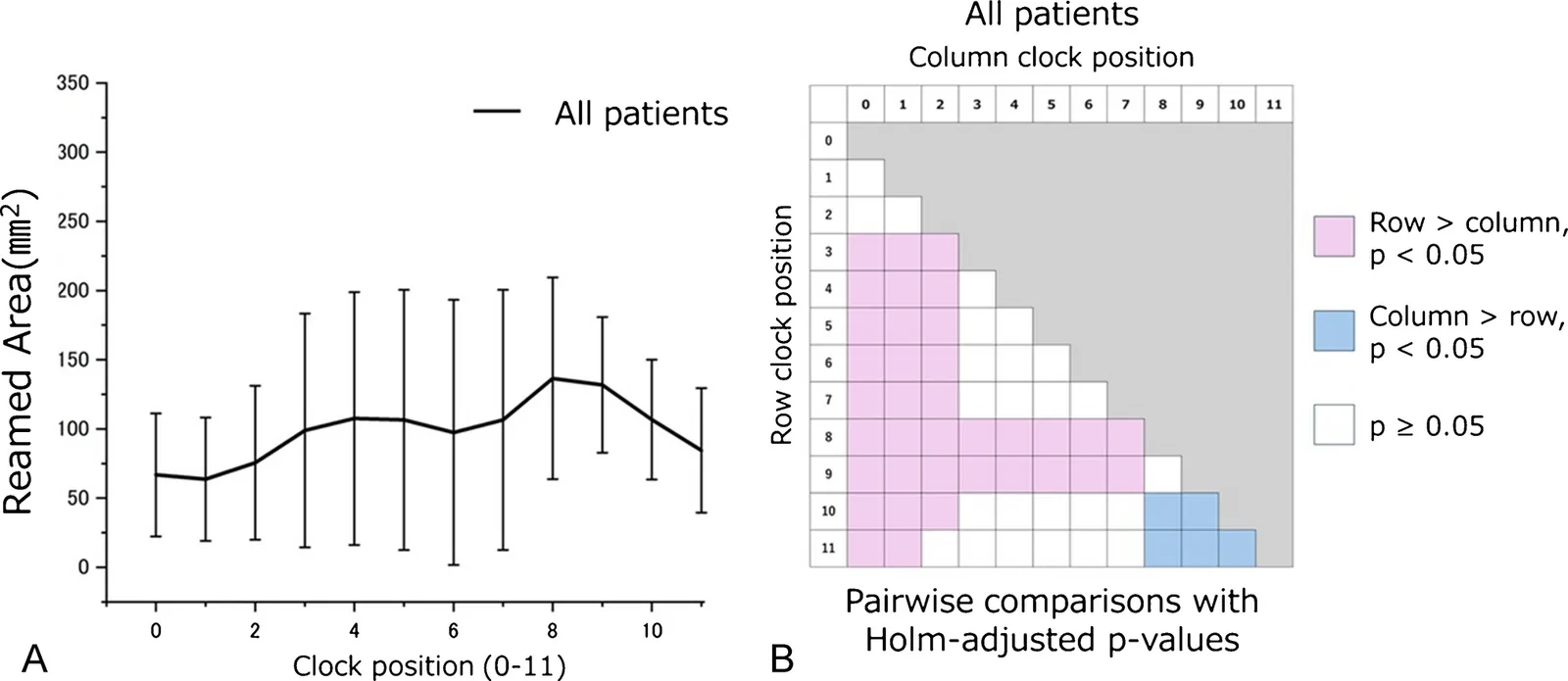
Clock-face distribution of simulated reamed area in all patients. A line graph shows the mean simulated reamed area with standard deviation at each clock position from 0 to 11 o’clock, measured radially from the cup center (**A**). A heatmap illustrates pairwise comparisons of simulated reamed area between clock positions based on repeated-measures analysis of variance (Greenhouse–Geisser corrected when sphericity was violated) with Holm-adjusted *p* values (**B**). Rows and columns indicate clock positions (0–11); pink cells indicate positions where the row clock position had a larger simulated reamed area than the column position (*p* < 0.05), blue cells indicate smaller simulated reamed areas at the row position (*p* < 0.05), and white cells indicate no significant difference (*p* ≥ 0.05)

The mean simulated reamed area was smallest at 1 o’clock (64 ± 45 mm^2^) and increased toward the posterior region, reaching a peak at 8 o’clock (137 ± 73 mm^2^) (Fig. 3A). Post hoc pairwise comparisons between clock-face positions with Holm adjustment demonstrated that the reamed areas at 8 and 9 o’clock were greater than those at all other positions (*p* < 0.05), whereas the reamed areas from 0 to 2 o’clock were smaller than those at most other positions (*p* < 0.05) (Fig. 3B).

**Fig. 4:**
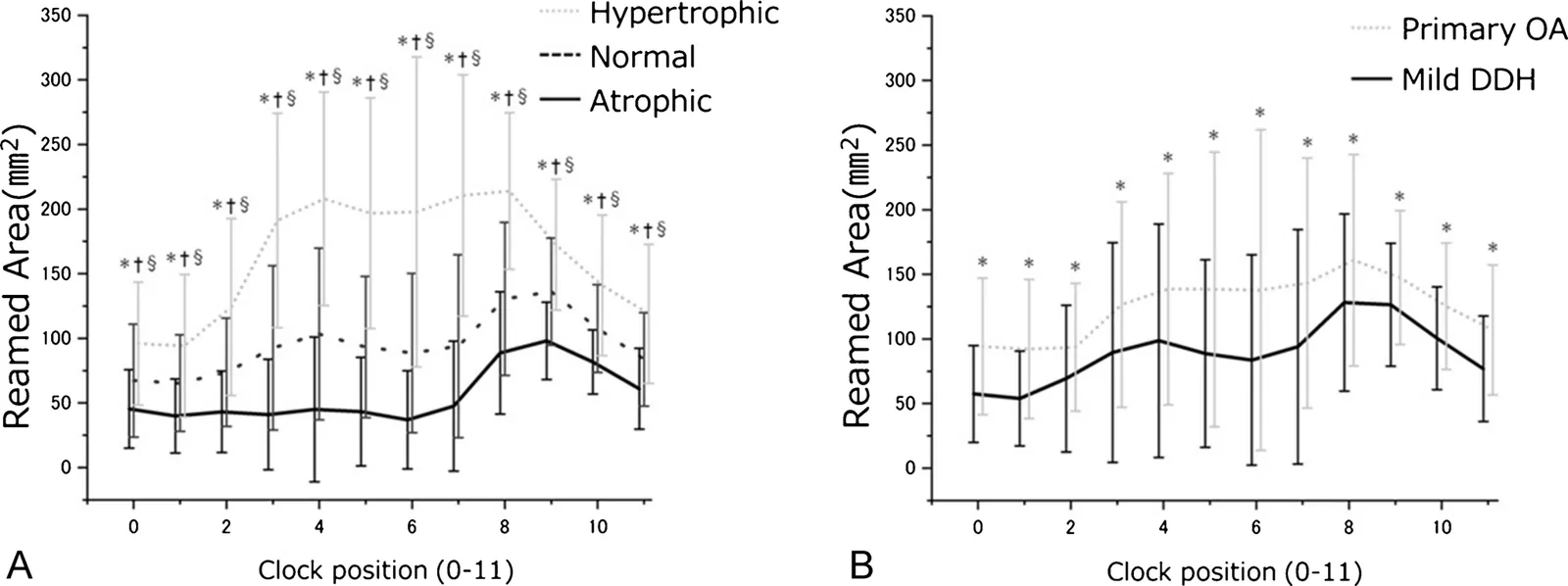
Distribution of simulated reamed area by OA type and diagnosis. Line graphs show the mean simulated reamed area with standard deviation at each clock position (0–11 o’clock) according to OA type based on the Bombelli classification (**A**) and according to the underlying diagnosis, primary OA versus mild DDH (**B**). In A, * atrophic vs. normal, †atrophic vs. hypertrophic, § normal vs. hypertrophic. In B, * primary OA vs. mild DDH. OA = osteoarthritis, DDH = developmental dysplasia of the hip

When OA-type distribution was compared between diagnoses, primary OA included fewer atrophic-type hips and more hypertrophic-type hips than mild DDH (Table 2). Simulated reamed areas were then evaluated according to OA type. For the total simulated reamed area across all clock positions, hypertrophic-type hips (1965 ± 570 mm^2^) exhibited the largest reamed area, followed by normal-type hips (1136 ± 482 mm^2^) and atrophic-type hips (672 ± 341 mm^2^) (p < 0.001). At all clock positions, hypertrophic-type hips showed larger reamed areas than normal-type hips, and normal-type hips showed larger reamed areas than atrophic-type hips (Fig. 4A). With respect to the underlying diagnosis, the total simulated reamed area across all clock positions was larger in hips with primary OA (1506 ± 736 mm^2^) than in those with mild DDH (1067 ± 618 mm^2^) (p = 0.002). Across all clock positions, hips with primary OA consistently demonstrated larger reamed areas than hips with mild DDH (Fig. 4B).

**Table 2:** Distribution of diagnosis and osteoarthritis type

	Primary osteoarthritis (*n*=30)	Mild developmental dysplasia of the hip (*n*=88)	*p* value
Osteoarthritis type (Bombelli classification)			
Hypertrophic, n (%)	13 (43.3)	16 (18.2)	
Normal, n (%)	14 (46.7)	34 (38.6)	
Atrophic, n (%)	3 (10.0)	38 (43.2)	
Total, n	30	88	< 0.001

Overall group comparison was performed using Fisher’s exact test. Post hoc pairwise comparisons of the proportions of each OA type between diagnoses were performed using Fisher’s exact tests with Holm correction: hypertrophic, *p* = 0.009; normal, *p* = 0.425; atrophic, *p* = 0.002

Regarding the directional distribution of simulated reamed areas, hypertrophic-, normal-, and atrophic-type hips showed distinct patterns: in hypertrophic-type hips, reamed areas were larger from 2 to 10 o’clock than at most other positions; in normal-type hips, larger reamed areas were observed at 8 and 9 o’clock; and in atrophic-type hips, reamed areas were larger from 8 to 10 o’clock (Supplementary Fig. 2). When stratified by diagnosis, primary OA showed greater reamed areas at 8 and 9 o’clock than at the superior positions from 11 to 2 o’clock (Supplementary Fig. 3). In mild DDH, reamed areas at 0–2 o’clock were smaller than those at most other clock positions, whereas those at 8 and 9 o’clock were larger than those at most other positions.

## Discussion

Appropriate acetabular reaming in cementless total hip arthroplasty (THA) is important for achieving stable press-fit fixation. However, the three-dimensional distribution of intra-acetabular reaming according to osteophyte morphology and underlying diagnosis remains unclear. In this CT-based simulation study, we quantified simulated reamed areas after standardized virtual cup placement at the true acetabulum. The estimated bone area that would require reaming was concentrated posteriorly and limited in the anterosuperior region. Hypertrophic-type hips showed the largest simulated reamed area, whereas mild DDH showed a smaller circumferential simulated reamed area than primary OA.

In the overall cohort, simulated intra-acetabular reaming showed substantial interpatient variability, with smaller areas from 0 to 2 o’clock and larger areas at 8 and 9 o’clock. Although a learning curve for intra-acetabular reaming has been reported [10, 11], training in acetabular reaming largely depends on the experience of supervising surgeons, and evidence-based guidance remains limited. Although textbooks/teaching resources often describe acetabular reaming as being directed medially and posteriorly [24], quantitative evidence supporting this concept remains limited. Anatomically, because the acetabular fossa is located slightly anterior to the acetabulum centre [25], greater posterior than anterior removal of subchondral bone would be expected. In addition, the slightly elliptical shape of the acetabular roof in the craniocaudal direction likely reduces the need for superior reaming when placing the cup at the true acetabulum [26]. These findings may help surgeons recognize that, during standardized cup placement, the superior-to-anterosuperior region has relatively limited bone overlap with the planned cup, suggesting that unnecessary over-reaming in this region should be avoided. The substantial variability in simulated reamed areas further underscores the importance of understanding how estimated direction-specific reaming patterns differ according to OA type and underlying diagnosis.

When evaluated by OA type, hypertrophic hips showed the largest simulated reamed area, followed by normal- and atrophic-type hips. Because the Bombelli classification reflects reparative responses such as osteophyte formation and subchondral sclerosis [12], this result is consistent with its conceptual basis. The similar pattern across all acetabular directions also suggests that intra-acetabular osteophyte formation in OA may occur circumferentially rather than being confined to specific regions. Primary OA showed greater simulated reaming than mild DDH, possibly reflecting the lower proportion of atrophic-type hips in primary OA. Although previous studies have reported more extensive acetabular osteophyte formation in severe DDH than in primary OA [27–29], our cohort was limited to mild DDH cases in which cup placement at the true acetabulum was feasible. Because greater femoral head displacement is associated with increased osteophyte formation [13], mild DDH likely includes more atrophic-type hips with less osteophyte formation. Consequently, mild DDH may exhibit a smaller circumferential reamed area, suggesting that surgeons should pay particular attention to the limited estimated bone-overlap area during preoperative planning.

**Fig. 5:**
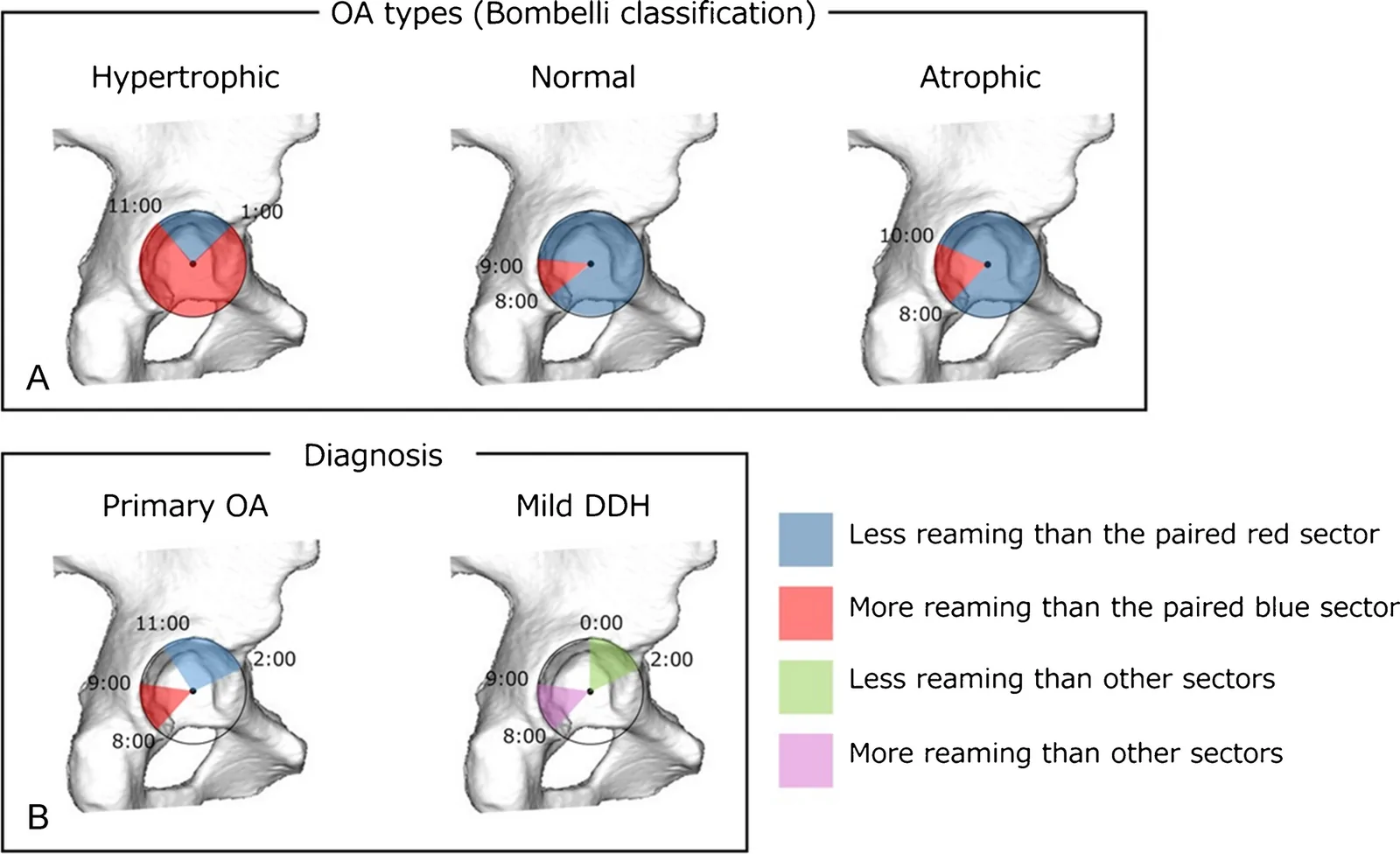
Schematic three-dimensional distribution of acetabular reaming according to OA type and underlying diagnosis. The acetabulum is shown in the functional pelvic plane using a clock-face system (0 o’clock cranial, with numbers increasing anteriorly). Blue sectors indicate regions with less reaming than the paired red sector within the same OA type. Green sectors indicate regions with less reaming than other sectors within the same diagnosis, whereas purple sectors indicate regions with more reaming than other sectors. (**A**) Distribution of reaming according to OA type based on the Bombelli classification (hypertrophic, normal, and atrophic). (**B**) Distribution of reaming according to the underlying diagnosis (primary OA and mild DDH). OA = osteoarthritis, DDH = developmental dysplasia of the hip

When simulated reamed areas at each clock-face position were compared by OA type, normal- and atrophic-type hips showed greater reamed areas in the posterior acetabulum than at other positions. In contrast, hypertrophic hips showed greater simulated reaming across the inferior acetabulum than in the superior acetabulum (Fig. 5A). This pattern likely reflects extensive osteophyte formation and pronounced double-floor formation as OA progresses [30]. Osteophytes within the acetabulum are known to begin forming around the acetabular fossa even in early-stage OA [31], and marked double-floor formation in hypertrophic OA may mislead surgeons when determining the appropriate cup position [32]. Therefore, in hypertrophic OA, careful recognition of double-floor formation may be important when planning cup placement and estimating the acetabular bone area requiring reaming. When simulated reamed areas were compared by underlying diagnosis, both primary OA and mild DDH showed larger areas in the posterior acetabulum than in other regions (Fig. 5B). In primary OA, however, this difference reached statistical significance only when compared with the superior to anterosuperior acetabulum, whereas in mild DDH, simulated reamed areas in the superior to anterosuperior acetabulum were smaller than at many other positions. Overall, mild DDH showed a smaller circumferential simulated reamed area than primary OA, which may partly reflect the higher proportion of atrophic-type hips in mild DDH. However, diagnosis-specific acetabular morphology may also contribute to this difference. Because the overall simulated reamed area was smaller in mild DDH, the estimated regions requiring reaming were more limited, making the posterior concentration of the simulated pattern more apparent. Therefore, in mild DDH, the simulated findings may help surgeons anticipate a more posteriorly concentrated estimated bone-overlap area while recognizing the relatively limited overlap in the anterosuperior region during preoperative planning.

### Limitations

This study has some limitations. First, this was a CT-based simulation study and did not evaluate actual intraoperative reaming, achieved postoperative cup position, functional cup orientation, daily postural changes, or clinical outcomes. The simulation also simplified reaming as linear advancement along a fixed insertion axis and did not replicate intraoperative adjustments. Therefore, the simulated reamed area should not be interpreted as a direct measure of surgical behaviour or as evidence that a specific reaming pattern improves fixation or clinical outcomes. Rather, the present findings represent a standardized anatomical estimate of bone overlap between the native acetabulum and a virtually placed cup. Future studies comparing simulated and achieved cup positions, intraoperative reaming patterns, daily postural changes, and postoperative outcomes are needed to validate the clinical implications of this model. Second, reaming was quantified as two-dimensional areas rather than three-dimensional volumes. Although volumetric assessment may provide additional information, area-based measures more intuitively illustrate directional differences. Third, hips with severe DDH and those with a simulated cup CE angle < 0° were excluded; thus, reaming patterns in cases requiring alternative strategies such as high hip centre placement, peripheral reaming without medialization [33], or structural bone grafting were not evaluated. Fourth, cartilage removal was not directly assessed; however, fibrocartilage in OA is thought to degenerate and be replaced by bone [34], suggesting that reamed areas may roughly approximate the distribution of tissue removal. Fifth, the diameter of the virtual cup differed among hips, and larger body size could inherently result in larger reamed areas. As in previous reports [35], body mass index differed between the mild DDH and primary OA groups; however, the mean cup diameter did not differ significantly, suggesting that the influence of body size on between-group comparisons was limited. Finally, the cohort consisted exclusively of Asian females. Because sex- and ethnicity-related differences in body size and acetabular morphology have been reported [18, 36, 37], the present findings should be generalized to male and non-Asian populations with caution. However, females with DDH account for most THA cases in Japan and other East Asian countries [38, 39], making this cohort representative of routine clinical practice in these regions.

## Conclusions

In this CT-based simulation study, standardized virtual cup placement at the true acetabulum demonstrated a posteriorly concentrated distribution of the estimated acetabular bone area requiring reaming, with relatively limited overlap in the superior-to-anterosuperior region. Mild DDH showed a smaller total estimated area but a clearer direction-specific pattern than primary OA. These findings may provide a quantitative anatomical reference for understanding direction-specific acetabular reaming patterns during preoperative planning and surgical education.

## Supplementary Information

Below is the link to the electronic supplementary material.Supplementary file1 (DOCX 4728 KB)

## Data Availability

No datasets were generated or analysed during the current study.
