# Supplementary material for: Where is acetabular reaming concentrated during total hip arthroplasty for hip osteoarthritis? A computed tomography-based simulation analysis
Source: Int Orthop. 2026 Jun 24;50(8):2103–11. doi: 10.1007/s00264-026-06901-4 (PMC13524963; doi:10.1007/s00264-026-06901-4)
Supplement: Supplementary file 1 [file 264_2026_6901_MOESM1_ESM.docx]

Supplementary Fig. 1 STROBE flow diagram

THA = total hip arthroplasty

Supplementary Fig. 2 Clock-face comparisons of reamed area by OA type

Heatmaps illustrate pairwise comparisons of reamed area between clock positions for hypertrophic, normal, and atrophic osteoarthritis types based on repeated-measures analysis of variance (Greenhouse–Geisser corrected when sphericity was violated) with Holm-adjusted p values. Rows and columns indicate clock positions (0–11); pink cells indicate positions where the row clock position had a larger reamed area than the column position (p < 0.05), blue cells indicate smaller reamed areas at the row position (p < 0.05), and white cells indicate no significant difference (p ≥ 0.05).

Supplementary Fig. 3 Clock-face comparisons of reamed area by diagnosis

Heatmaps illustrate pairwise comparisons of reamed area between clock positions for primary osteoarthritis and mild developmental dysplasia of the hip based on repeated-measures analysis of variance (Greenhouse–Geisser corrected when sphericity was violated) with Holm-adjusted p values. Rows and columns indicate clock positions (0–11); pink cells indicate positions where the row clock position had a larger reamed area than the column position (p < 0.05), blue cells indicate smaller reamed areas at the row position (p < 0.05), and white cells indicate no significant difference (p ≥ 0.05).
